# Exploring the neuroprotective potential of naringin following spinal cord injury in rats: improving sensory and motor function through combating inflammation and oxidative stress

**DOI:** 10.3389/fphar.2025.1545049

**Published:** 2025-04-28

**Authors:** Fatemeh Moradi, Sajad Fakhri, Amir Kiani, Fatemeh Abbaszadeh, Mohammad Hosein Farzaei, Javier Echeverría

**Affiliations:** ^1^ Student Research Committee, Kermanshah University of Medical Sciences, Kermanshah, Iran; ^2^ Pharmaceutical Sciences Research Center, Health Institute, Kermanshah University of Medical Sciences, Kermanshah, Iran; ^3^ Regenerative Medicine Research Center, Health Technology Institute, Kermanshah University of Medical Sciences, Kermanshah, Iran; ^4^ Neurobiology Research Center, Institute of Neuroscience and Cognition, Shahid Beheshti University of Medical Sciences, Tehran, Iran; ^5^ Departamento de Ciencias del Ambiente, Facultad de Química y Biología, Universidad de Santiago de Chile, Santiago, Chile

**Keywords:** spinal cord injury, naringin, neuropathic pain, inflammation, motor dysfunction, oxidative stress

## Abstract

**Introduction:**

Spinal cord injury (SCI) leads to widespread cascades of inflammatory and oxidative factors. This pathological condition damages nerves and causes neurological disorders. To address these complex conditions, it is important to identify therapeutic candidates that affect multiple dysregulated signaling mediators and targets. Some phytochemicals such as naringin (NAI) with neuroprotective, antioxidant, and anti-inflammatory effects can be seen as a possible candidate for treating neurodegenerative diseases.

**Purpose:**

Therefore, this study aims to evaluate the impact and mechanism of NAI on sensory and motor function in rats with SCI.

**Materials and methods:**

In total, 35 rats were studied in five groups, including sham, SCI, and three groups treated with intrathecal administration of NAI (5, 10, and 15 mM). After the injury, sensorimotor behavioral tests and weight changes were performed for 4 weeks. On the 28^th^ day, the serum of rats was checked to measure biochemical factors such as catalase, glutathione, and nitrite and the activity of metalloproteinases 2 (MMP-2) and MMP-9. Also, histological changes in spinal cord tissue were evaluated weekly for 4 weeks.

**Results and discussion:**

NAI treatment demonstrated significant benefits in rats with SCI, including reducing pain, improvement in motor performance, and attenuated animal weight gain. Besides, NAI decreased the lesion area of spinal tissue and enhanced neuronal survival at both ventral and dorsal horns of spinal tissue. Furthermore, serum analysis revealed that NAI increased MMP-2 activity and catalase and glutathione levels while decreasing nitrite and MMP-9 activity.

**Conclusion:**

The intrathecal administration of NAI can be proposed as a proper alternative in the treatment of sensory-motor disorders caused by SCI through neuroprotective, anti-inflammatory, and antioxidant mechanisms.

## 1 Introduction

Spinal cord injury (SCI) significantly impairs an individual’s performance, quality of life, and social independence. There are two classes of SCI; traumatic and non-traumatic. An external physical trauma typically causes traumatic SCI and it is more prevalent (Ahuja et al., 2017), and non-traumatic SCI (New and Biering-Sørensen, 2017), is caused by degenerative, inflammatory, neoplastic, and infectious triggers (Buzzell et al., 2019; Musubire et al., 2019). Depending on the size and location of SCI, it can result in partial or complete paralysis, sensory dysfunctionality, bowel/bladder incontinence, and autonomic impairment (Schuld et al., 2016; Carrasco et al., 2018). The pathophysiology of SCI can be segmented into two phases. The first phase involves immediate impacts on the spinal cord, while a range of biochemical cascades marks the progressive phase. Notably, oxidative stress and inflammation play crucial roles in the development of tissue damage during this stage (Alizadeh et al., 2015; Carrasco et al., 2018; Hayta and Elden, 2018). The primary source of reactive oxygen species (ROS) after SCI is the activation of immune cells, such as microglia and infiltrating macrophages. These cells release pro-inflammatory cytokines, chemokines, and free radicals, leading to tissue damage and cell death. Oxidative stress further exacerbates the inflammatory response by activating transcription factors like nuclear factor kappa B (NF-κB). NF-κB promotes the production of additional pro-inflammatory mediators, amplifying the inflammatory cascade. Inflammation, in turn, contributes to oxidative stress by increasing the production of ROS and impairing the antioxidant defense mechanisms. After SCI, inflammatory cells by releasing matrix metalloproteinases (MMPs) can lead to the activation of inducible nitric oxide synthase (iNOS), which in turn can lead to the production of ROS. These processes further contribute to tissue damage, neuronal cell death, and functional impairment after SCI (Hellenbrand et al., 2021; Freyermuth-Trujillo et al., 2022). Accordingly, there is a complex pathophysiological mechanism behind SCI, which urges the need for finding novel multi-targeting agents. Hence, it appears that interventions aimed at mitigating oxidative stress and inflammatory factors have the potential to enhance the wellbeing of individuals with SCI.

Some phytochemicals have shown a bright future in combating neurodegenerative diseases by targeting multiple mechanisms. Naringin (NAI) is a beneficial glycoside flavonoid concentrated from the peel of citrus fruits and causes its bitter taste. NAI has been reported to have strong antioxidant, anti-inflammatory, anti-viral, anti-diabetic, and anticancer properties (Emran et al., 2022; Stabrauskiene et al., 2022). According to the research, this component can cross the blood-brain barrier and protect brain tissue by modulating brain chemistry (Sachdeva et al., 2014; Hassan et al., 2022). In terms of mechanism, NAI plays critical roles in various signaling pathways in degenerative diseases through modulating angiogenesis (Kandhare et al., 2014), inhibiting apoptotic proteins such as caspases and Bcl-2 family members, and activating prosurvival pathways like the phosphatidylinositol 3-kinase (PI3K)/protein kinase B (Akt) pathway, which promotes cell survival and inhibits apoptosis (Wang et al., 2020). We also previously showed the potential of naringenin, the aglycone of naringin, in the improvement of neuropathic pain and motor dysfunction after SCI (Fakhri et al., 2022c). Particularly, there is no FDA-approved drug against SCI. While some reports suggested that methylprednisolone may cause neuroprotective effects, the drug has shown to be ineffective in reducing neurological impairment and could even worsen spinal tissue damage, leading to secondary side effects (Evaniew et al., 2015; Jongen et al., 2016).

Considering the previous neuroprotective potentials of NAI in other neurodegenerative disease and the naringenin (NAI aglycone) neuroprotection potential against SCI complications, along with the absence of any FDA-approved drug in SCI, this study aimed to assess the effect of intrathecal (i.t.) administration of NAI on motor and sensory impairments after SCI in rats, unveiling its neuroprotective, anti-inflammatory, and antioxidant effects.

## 2 Materials and methods

### 2.1 Chemicals

Naringin was obtained from Sigma-Aldrich (Sigma Chemical Co., St. Louis, MO, United States), while cefazolin was sourced from Exir Company (Iran). Additionally, ketamine and xylazine were purchased from Alfasan (Alfasan IBV, Turkey). All other chemicals and reagents used were of analytical reagent grade and procured from commercial suppliers.

### 2.2 Animals

For this study, we recruited 35 adult male Wistar rats, 230–250 g, from the reproductive colony at Kermanshah University of Medical Sciences. The rats were housed in an environment optimized, featuring a controlled 24-h cycle (light 12 h and dark 12 h) and a consistent temperature of around 24°C. This study is based on the guidelines of the National Institutes of Health regarding laboratory care and use approved by the Ethics Committee for Working with Laboratory Animals in Kermanshah University of Medical Sciences (IR.KUMS.REC.1400.417). Each rat was housed in separate clean cages with water and food *ad libitum* for further experimental studies.

### 2.3 Experimental groups

In total, 35 rats were placed in 5 groups of 7, including sham, SCI, and NAI-treated with three different doses, obtained from relative derivatives (Fakhri et al., 2022c). The sham group underwent laminectomy without any compressive injury. The SCI group experienced a compressive injury. The remaining three groups also were subjected to i.t. administration of NAI (5, 10, and 15 mM, 10 μL) 30 min after the compressive injury. Sensory and motor behavioral tests were performed on animals before surgery and every week from days 7–28 after surgery. On day 28, serum and spinal cord tissue samples were collected and analyzed for molecular and histopathological changes.

### 2.4 Spinal cord injury

A mixture of ketamine and xylazine at a dosage of 60/10 mg/kg (Fakhri et al., 2022c) was utilized to anesthetize the rats. Vertebral bone removal (laminectomy) was performed at the level of the 8^th^ and 9^th^ vertebrae of the thoracic region (T8-T9) using tiny rongeur (Fine Science Tools, United States). Then, the spinal cord tissue was compressed with an aneurysm clip applying a force of 90 g (Aesculap, Tuttlingen, Germany) for 1 minute (Fakhri et al., 2018; 2019). During the surgery, rats were anesthetized and lidocaine/epinephrine was used for injection around T8-T9 to avoid possible pain/excessive bleeding sensation. The skin and muscles were then sutured. After surgery, the rats received 40 mg/kg of intramuscular cefazolin to reduce the risk of infection and subcutaneous injection of normal saline (2 mL) to ensure rehydration. Until the bladder reflex returned, animals’ bladders were emptied manually twice in a day (Ahmadpour et al., 2025).

### 2.5 NAI injection

I.t. Injection was done using the method of Mestre (Mestre et al., 1994). To perform the injection, a Hamilton syringe was utilized, which was linked to a 25-gauge needle through a 10-diameter polyethylene tube. The needle was carefully placed at an angle of 45° to the spinal cord in the gap between lumbar 6 (L6) and L5. Once the tail reflex was observed, confirming the accurate injection site, the drug was then gradually administered into the subarachnoid space.

### 2.6 Behavioral assessments

#### 2.6.1 Acetone drop test

For evaluation of cold allodynia, rats were placed in a mesh chamber and a drop of acetone was applied to the plantar surface of the hind paw. The rapid evaporation of acetone creates a feeling of cold and aversive behavior in animals suffering from cold allodynia. The severity of these reactions is quantified using a 0–4 scale, where 0 indicates no response, 1 represents a startled reaction without withdrawal, 2 signifies partial withdrawal, 3 denotes prolonged and repeated withdrawal, and 4 is characterized by paw licking and flapping (Fakhri et al., 2018).

#### 2.6.2 Hot plate test

The hot plate is a plate heated by an electric current. This test is a widely used method to evaluate hyperalgesia in rats. In the experiment, the rats were placed on a heated surface with a temperature of 50 ± 2°C. The time taken for the rats to lift their paw or jump from the hot plate was measured as the response latency to the thermal stimulus with a cut-off of 60 s (Fakhri et al., 2022c).

#### 2.6.3 Von Frey test

The von Frey test was employed to assess mechanical allodynia by applying a series of thin filaments with varying forces to the paw surface between the second and third toes. Each filament was applied five times to evaluate sensitivity. If the animal responded to a filament three times, it was noted as a reaction. Paw withdrawal, tremors, and vocalizations were regarded as indications of mechanical allodynia (Bagheri Bavandpouri et al., 2024).

#### 2.6.4 BBB test

Basso, Beattie, and Bresnahan (BBB) motor evaluation methods were performed to determine motor behavior. The movements of the animals were assessed in a box with a surface area of 90 cm^2^ and walls of around 10 cm for 4 min. The BBB scale was determined in the range zero (complete paralysis of the hind limbs) to 21 (normal gait). The average scores of both paws were recorded as responses (Fakhri et al., 2021).

#### 2.6.5 Inclined plane test

The inclined plane test is a behavioral test in rats to assess hind limb motor deficits and muscle strength. The rats were positioned on an adjustable inclined plane with angles ranging from 0 to 70°. The angle of the inclined plane was gradually increased until the rat was unable to maintain its position and slipped. The maximum angle at which to stay at least for 5 s was recorded as the “slip angle” (Fakhri et al., 2018).

#### 2.6.6 Weight changes

During 4 weeks, the weight of the rats was recorded weekly. Fluctuating animal weight changes for each group were obtained from the difference between the weight of the rats before the injury and the weight of the rats on each of the days after the surgery/injury (Fakhri et al., 2019).

### 2.7 Glutathione activity

To evaluate glutathione levels, we employed the Ellman technique, which relies on the interaction between glutathione and Ellman’s reagent, 5,5′-dithio-bis(2-nitrobenzoic acid) (DTNB). The experimental setup involved adding 50 µL of phosphate-buffered saline (PBS) at pH 7.4 and 60 µL of rat serum to each well. Subsequently, they incubated with 100 µL of DTNB at 37°C for 10 min. The optical density was read at a wavelength of 412 nm using a microplate reader (Eyer and Podhradský, 1986).

### 2.8 Catalase activity

To assess catalase activity, we utilized the Aebi method, which relies on the hydrogen peroxide assay. The procedure involved sequentially adding 20 μL of serum and 100 μL of 65 mM hydrogen peroxide to the wells, followed by a 4-min incubation at 25°C. The reaction was stopped by adding 100 μL of 32.4 mM ammonium molybdate. The final step involved measuring the concentrations of the yellow molybdate complex and hydrogen peroxide at 405 nm (Aebi, 1984).

At the end for both glutathione and catalase assays, the difference in absorption between the sham group and other groups (SCI or NAI) was calculated using the following formula:

$$\text{Concentration difference }\left(\%\right)=\left[\left(C_{\text{sham}}\;–\;C_{\text{sample}}\right)/C_{\text{sham}}\right]\times 100$$

### 2.9 Nitrite test

To assess the activity of nitrite, the Griess method was used. Initially, serum samples (100 µL) were mixed with sulfanilamide solution (50 μL, dissolved in 5% HCl) in plate wells and incubated in the dark for 5 minutes. Following this, naphthyl ethylene diamine dihydrochloride (NEDD; 0.1% in distilled water) was added in a volume of 50 μL, and the plate was incubated in the dark at 37°C for 30 minutes. Optical density measurements were taken at 540 nm. In parallel, a standard curve was constructed using sodium nitrite at various concentrations (100 µL each) (Sun et al., 2003).

### 2.10 Zymography

The enzymatic activities of MMP-2 and MMP-9 were evaluated using gelatin zymography. This involved loading 100 μg of total serum protein into each sample. The electrophoretic separation was performed under a constant voltage of 150 V. Post-electrophoresis, the gels underwent renaturation in a Triton X-100 and Tris-HCl buffer (pH 7.5) on a shaker. Next, the gels were incubated for 18 h at 37°C in a buffer containing CaCl_2_, NaN_3_, and NaCl dissolved in Tris-HCl. After staining with Coomassie blue, the gels were destained using a mixture of acetic acid and methanol. The band intensities were quantified using ImageJ software (Hashemi et al., 2024).

### 2.11 Histological analysis

For hematoxylin and eosin (H&E) staining of spinal cord tissue, the animals were first perfused with phosphate-buffered saline (PBS) and paraformaldehyde. Subsequently, a 1-cm section of the spinal cord tissue, centered on the injury site, was isolated. After undergoing processing in various alcohols and embedding in a paraffin block, the tissue was sliced to a thickness of 4 μm. Following this, H&E dye was used to stain the tissue. Images of the stained sections were captured using a Nikon E600 optical microscope at magnifications of ×4 and ×40. The ImageJ software was then utilized to analyze the lesion size and count the number of neurons present in the spinal cord (Bagheri Bavandpouri et al., 2024). The lesion area was calculated as per the following formula:

Lesion area: total area of spinal cord - area of spared myelin/total area of spinal cord × 100.

### 2.12 Statistical analysis

The obtained data was analyzed using version 9.0 of GraphPad Prism. A two-way analysis of variance with Bonferroni *post hoc* was employed to evaluate the behavioral results, while histological and biochemical outcomes were assessed using a one-way analysis of variance with Tukey *post hoc*. A significance level of *p* < 0.05 was used for all calculations. All data were assessed as mean ± standard error of the mean (SEM).

## 3 Results

### 3.1 Behavioral assays

#### 3.1.1 NAI reduced cold allodynia after SCI

**FIGURE 1:**
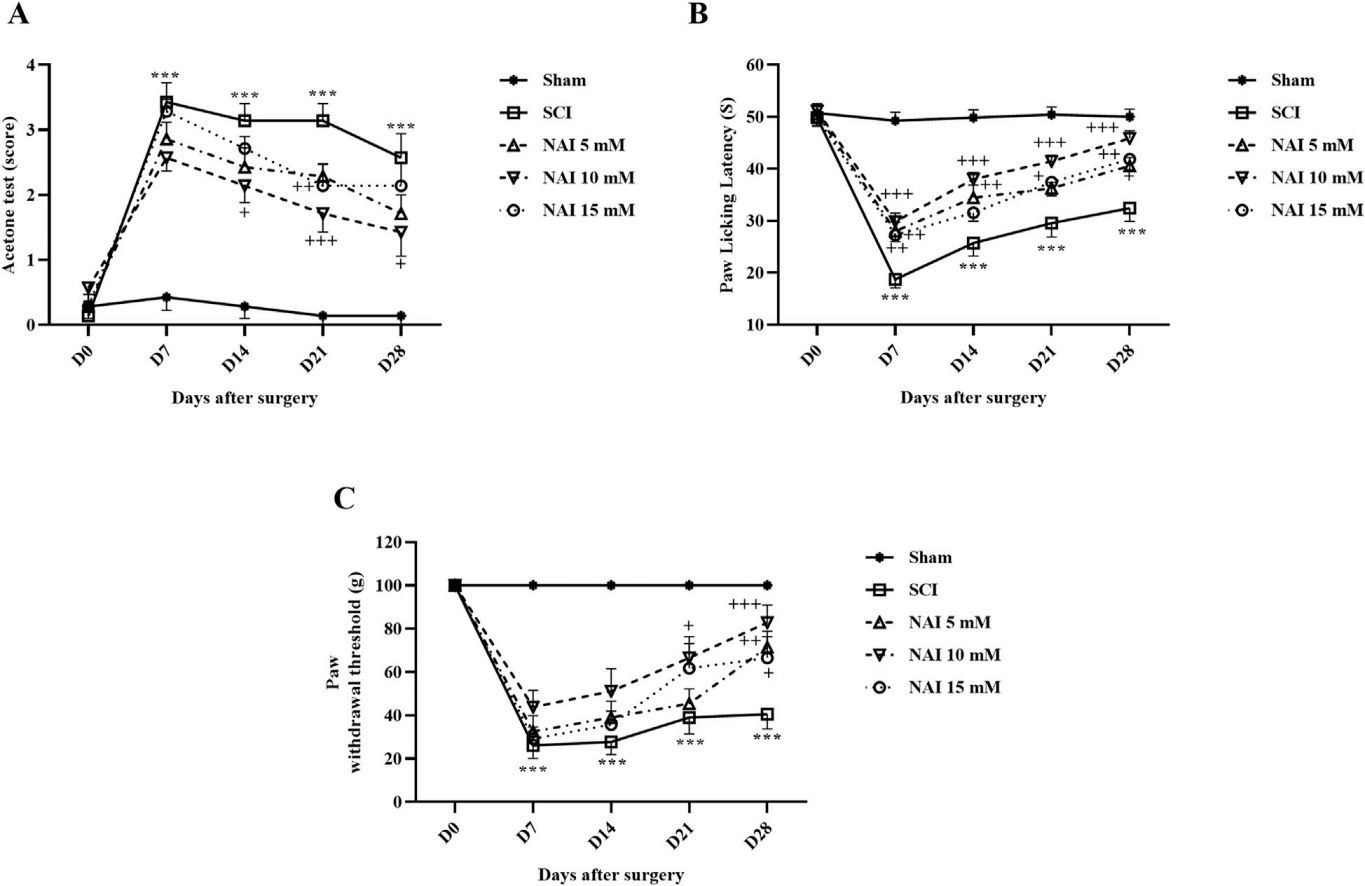
The impact of NAI on pain-related behaviors after compression SCI was assessed by measuring the paw response threshold to cold **(A)**, thermal **(B)**, and mechanical **(C)** stimuli. The data presented are expressed as mean ± SEM (*n* = 7). ^***^ *p* < 0.001 vs. sham group and ^+^ *p* < 0.05, ^++^ *p* < 0.01, ^+++^ *p* < 0.001 vs. SCI group. SCI: spinal cord injury; NAI: naringin (10 µL).

According to the obtained data (Figure 1A), it was observed that in the measurement of cold pain, the paw withdrawal reflex in the sham group remained unchanged with no response. However, after injury, the rats strongly reacted to the cold stimulus, indicating a significant difference compared to the sham group throughout the 4 weeks (*p* < 0.001). Furthermore, the group treated with NAI showed improved outcomes compared to the SCI group on the 14^th^, 21^st^, and 28^th^ days (*p* < 0.05). Notably, the most favorable results were observed in the group receiving NAI at a dose of 10 mM.

#### 3.1.2 NAI reduced heat hyperalgesia after SCI

The data analysis revealed that in the sham group, the delay in licking the paw (indicating pain threshold from heat) remained consistent throughout the entire experiment. However, in the SCI group, there was a notable decline in the delay time for licking the paw (*p* < 0.001). Additionally, when comparing the group treated with NAI, particularly at a 10 mM dose, to the SCI group, there was a meaningful elevation in the time taken to reach the pain threshold from the 7^th^ day until the 28^th^ day of treatment (*p* < 0.05) (Figure 1B).

#### 3.1.3 NAI reduced mechanical allodynia after SCI

The sensitivity to contact stimuli in the sham group remained relatively stable over 28 days. However, following SCI, there was a significant difference observed in response (increased sensitivity) to stimuli compared to the sham group (*p* < 0.001). Additionally, the groups treated with NAI (especially the 10 mM dose) exhibited notable improvements in performance during 28 days, which was a considerable difference compared to the SCI group (*p* < 0.05) (Figure 1C).

#### 3.1.4 NAI improved motor behavior after SCI

**FIGURE 2:**
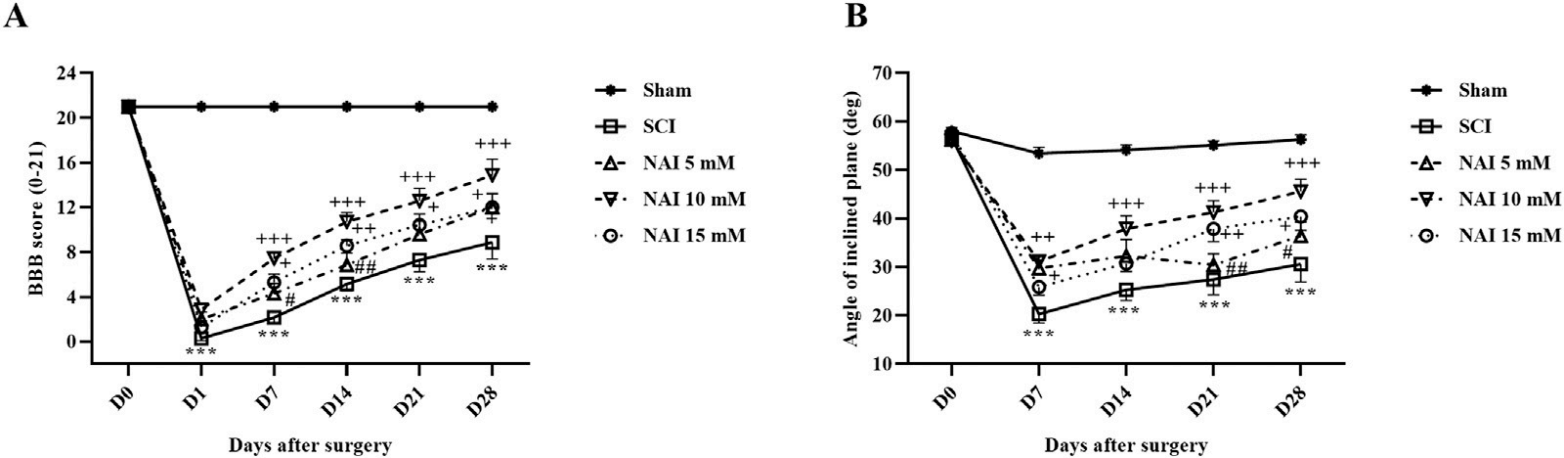
The impact of NAI on locomotor activity after compression SCI. Basso, Beattie, and Bresnahan (BBB) score **(A)** and the inclined plane test **(B)**. The data presented are expressed as mean ± SEM (*n* = 7). ^***^ *p* < 0.001 vs. sham group and ^+^ *p* < 0.05, ^++^ *p* < 0.01, ^+++^ *p* < 0.001 vs. SCI group and ^#^ *p* < 0.05, ^##^ *p* < 0.01, vs. NAI 10 mM group. SCI: spinal cord injury; NAI: naringin (10 µL).

Motor behavior was assessed following injury using the motor scale method known as the BBB (Figure 2A). The sham group did not show any changes in their BBB score (consistent score of 21), indicating no motor impairments after the laminectomy procedure. In contrast, the SCI groups exhibited significant decreases in motor performance compared to the sham group (*p* < 0.001). Treatment with NAI showed promising results in improving motor performance starting from the first week of treatment (*p* < 0.05). Among the different doses tested, the group treated with a 10 mM dose of NAI demonstrated the most substantial improvement (*p* < 0.05).

Besides, the findings of the inclined plane test indicated that the average resistance angle to falling (70°) remained unchanged in the control group throughout the study, suggesting no movement impairment following laminectomy (Figure 2B). However, the SCI group experienced a significant decrease in their ability to stand on the ramp, and by day 28, this reduction was significant compared to the sham group (*p* < 0.001). Conversely, treatment with varying doses of NAI, especially the 10 mM dose, enhanced the balance of the rats to stand on the ramp from the 7^th^ day after injury (*p* < 0.05).

### 3.2 NAI increased glutathione/catalase and decreased nitrite levels

**FIGURE 3:**
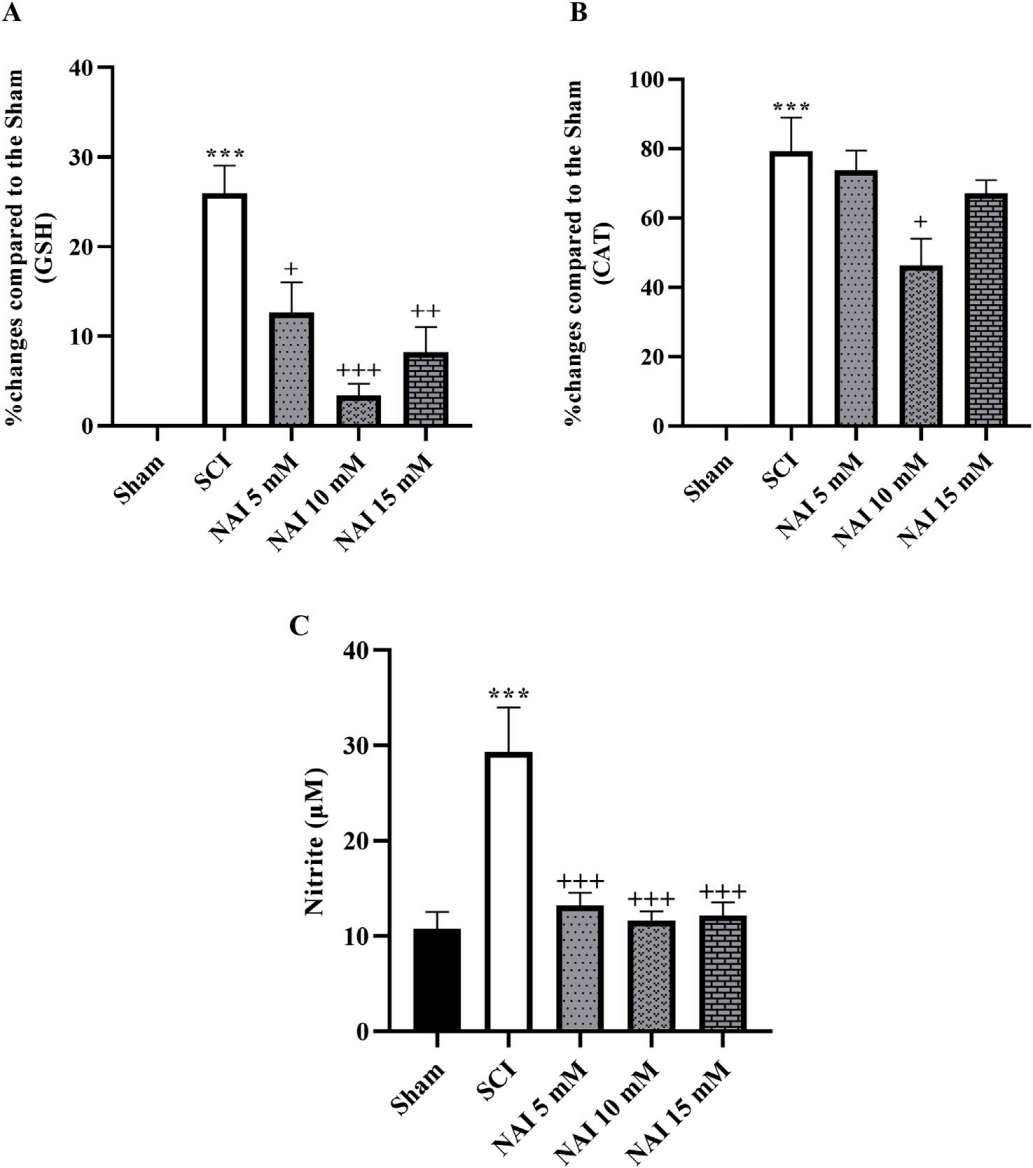
The impact of NAI on changes in serum levels of GSH **(A)** and CAT **(B)** and nitrite **(C)** level after compression SCI. The data presented are expressed as mean ± SEM (*n* = 3). ^***^ *p* < 0.001 vs. sham group and ^+^ *p* < 0.05, ^++^ *p* < 0.01, ^+++^p < 0.001 vs. SCI group. SCI: spinal cord injury; NAI: naringin (10 µL), GSH: glutathione; CAT: catalase.

The analysis conducted to measure the changes in glutathione (Figure 3A) and catalase (Figure 3B) revealed a substantial reduction in their levels after SCI (*p* < 0.001). However, treatment with NAI effectively compensated for this decrease in activity. Among the treatment groups, the highest increase in activity was observed in the group treated with a dose of 10 mM (*p* < 0.05). In addition, the SCI group exhibited a marked increase in nitrite levels compared to the sham group (*p* < 0.001, Figure 3C). The administration of NAI led to a remarkable decrease in nitrite levels compared to the SCI group (*p* < 0.001).

### 3.3 NAI increased the activity of MMP-2, while decreasing MMP-9 after SCI

**FIGURE 4:**
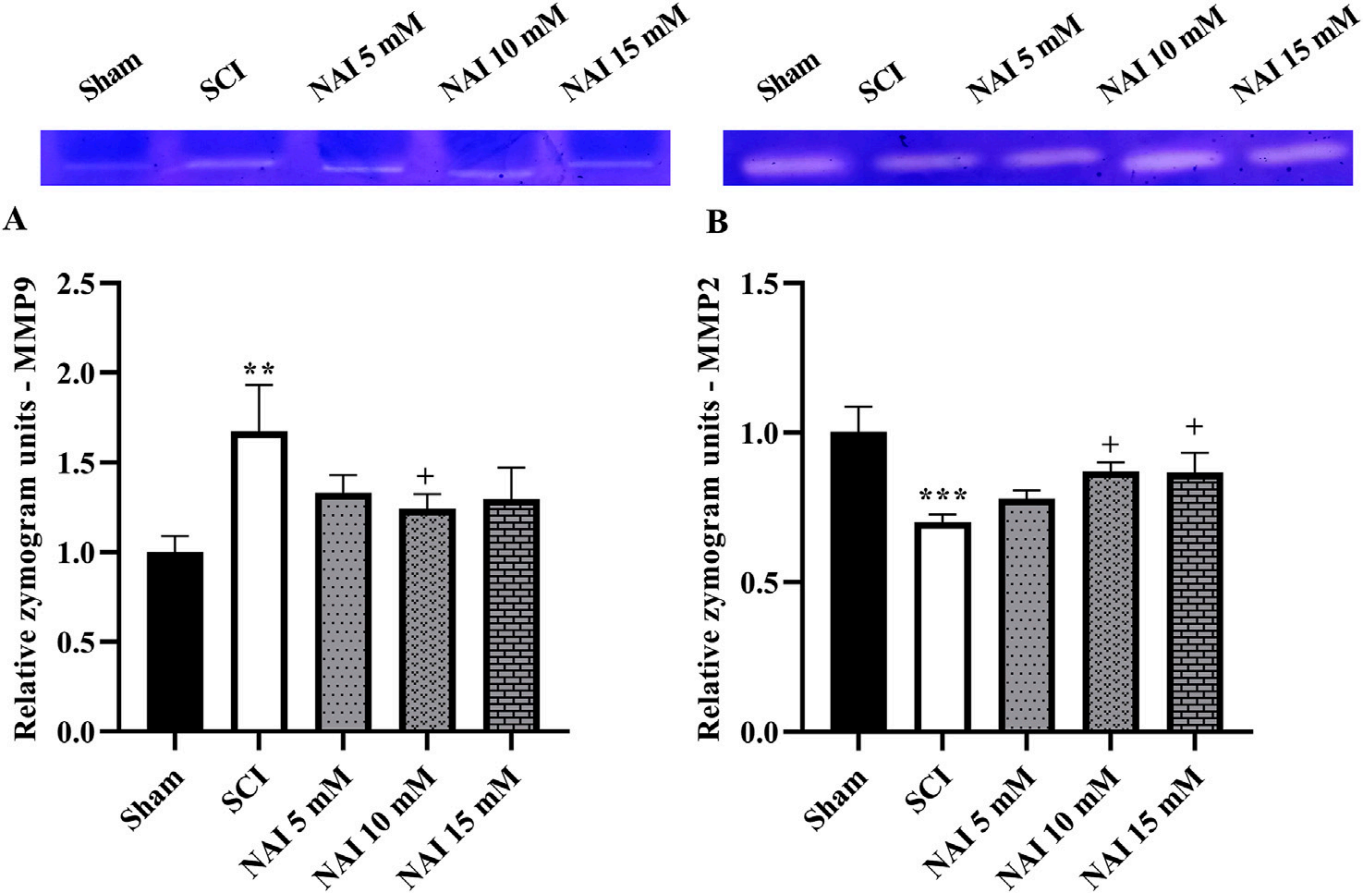
The impact of NAI on MMP-9 and MMP-2 activity after compression SCI. MMP-9 **(A)** and MMP-2 **(B)**. Data presented as mean ± SEM. ^**^ *p* < 0.01, ^***^ *p* < 0.001 vs. sham, ^+^ *p* < 0.05 vs. SCI group. MMP: metalloproteinases; SCI: spinal cord injury; NAI: naringin (10 µL).

Following injury, the SCI group showed an increase in MMP-9 (*p* < 0.01, Figure 4A) activity and a decrease in MMP-2 (*p* < 0.001, Figure 4B) activity when compared to the sham group. However, with NAI treatment, these alterations were effectively reversed and brought the levels of both MMP-2 and MMP-9 closer to their initial states (*p* < 0.05).

### 3.4 NAI decreased histopathological damage after SCI

**FIGURE 5:**
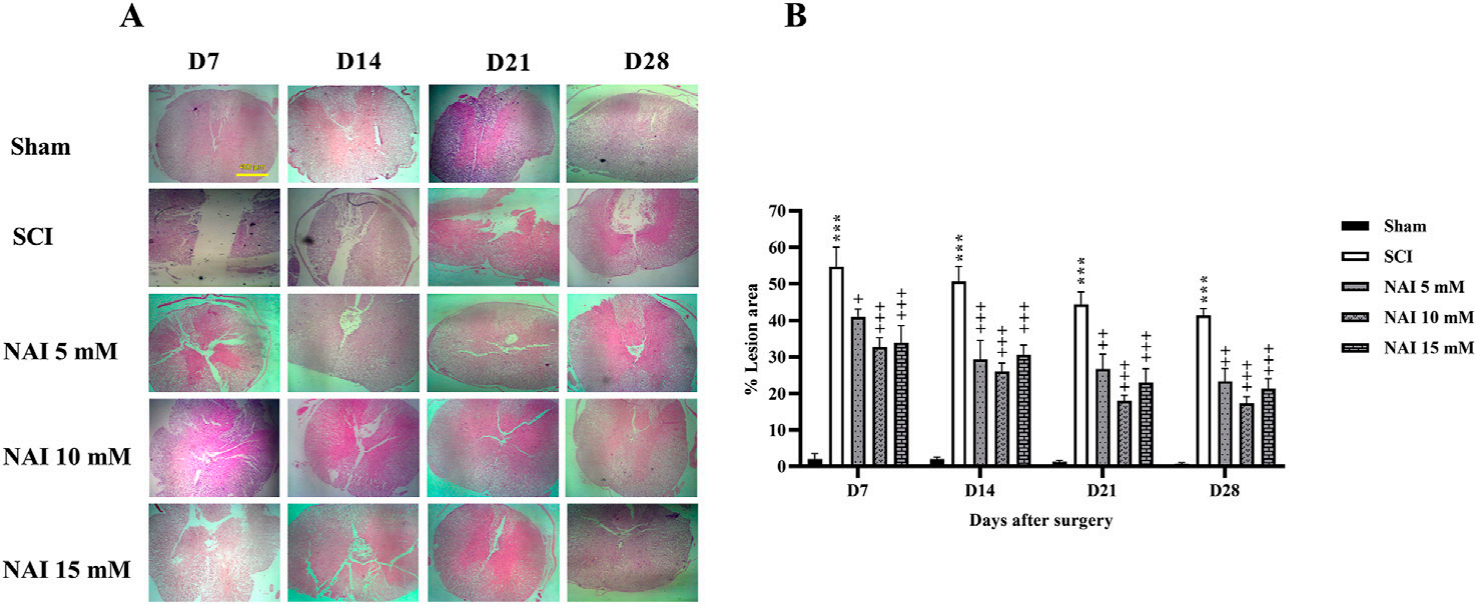
The impact of NAI on %lesion area on the spinal cord tissue after compression SCI. ×4 magnification of spinal cord **(A)**, and lesion area analysis **(B)**. The data presented are expressed as mean ± SEM. ^***^ *p* < 0.001 vs. sham group and ^+^ *p* < 0.05, ^++^ *p* < 0.01, ^+++^ *p* < 0.001 vs. SCI group. SCI: spinal cord injury; NAI: naringin (10 µL).

**FIGURE 6:**
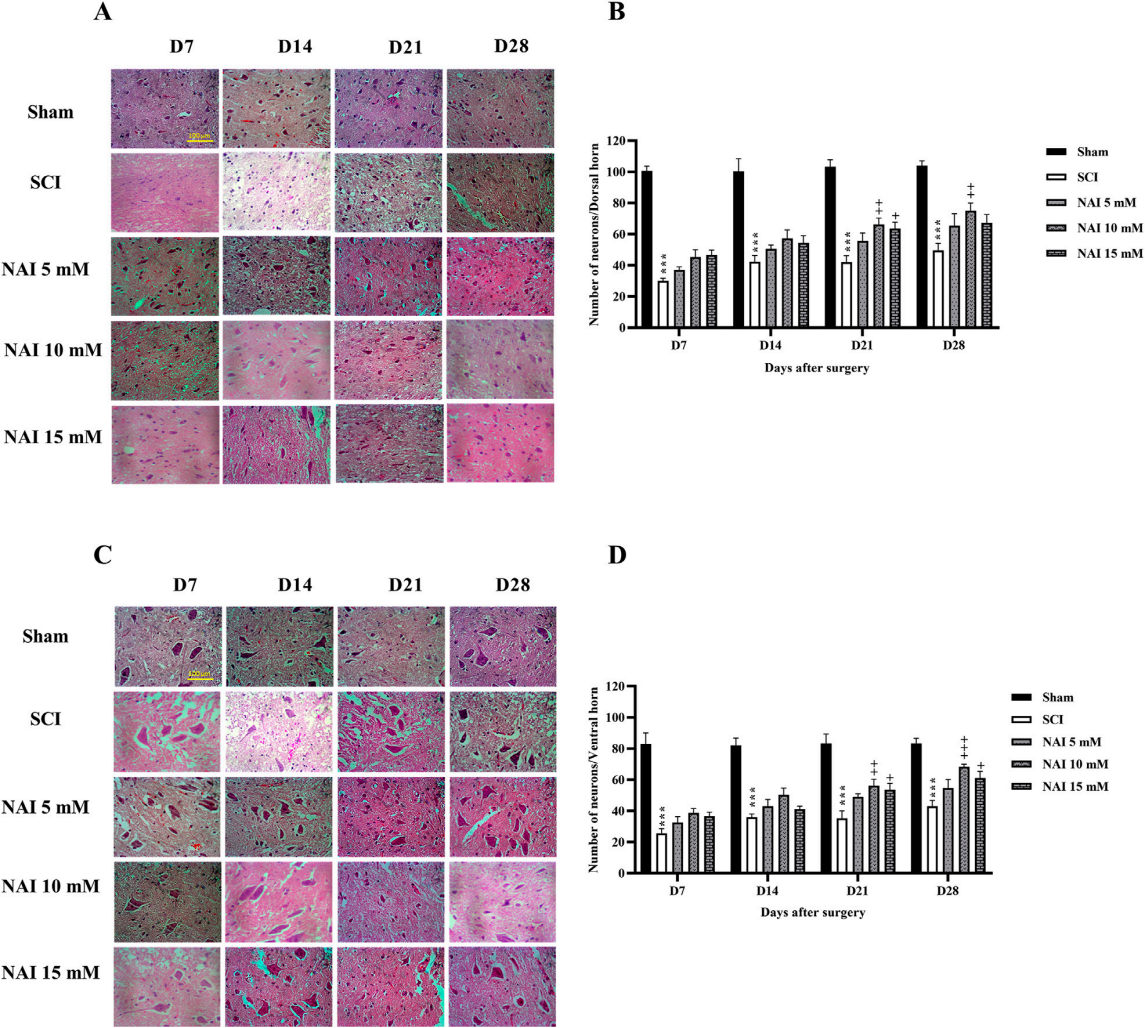
The impact of NAI on the number of neurons of the spinal cord after compression SCI. Dorsal **(A,B)**, and ventral **(C,D)** horns. The data presented are expressed as mean ± SEM. ^***^ *p* < 0.001 vs. sham group and ^+^ *p* < 0.05, ^++^ *p* < 0.01, ^+++^ *p* < 0.001 vs. SCI group. SCI: spinal cord injury; NAI: naringin (10 µL).

Figure 5 illustrates the use of H&E staining to assess tissue damage and histological changes. The findings demonstrate that the injury group had a larger damaged area compared to the sham group (*p* < 0.001, Figures 5A,B). This was accompanied by a reduction in the number of dorsal (Figures 6A,B) and ventral (Figures 6C,D) horn neurons (*p* < 0.001). In contrast, treatment with NAI effectively decreased the size of the injury site, prevented neuronal death, and preserved neurons. These beneficial effects were observed across all doses, particularly with the 10 mM dose, starting from the first week (*p* < 0.05).

### 3.5 NAI attenuated weight changes after SCI

**FIGURE 7:**
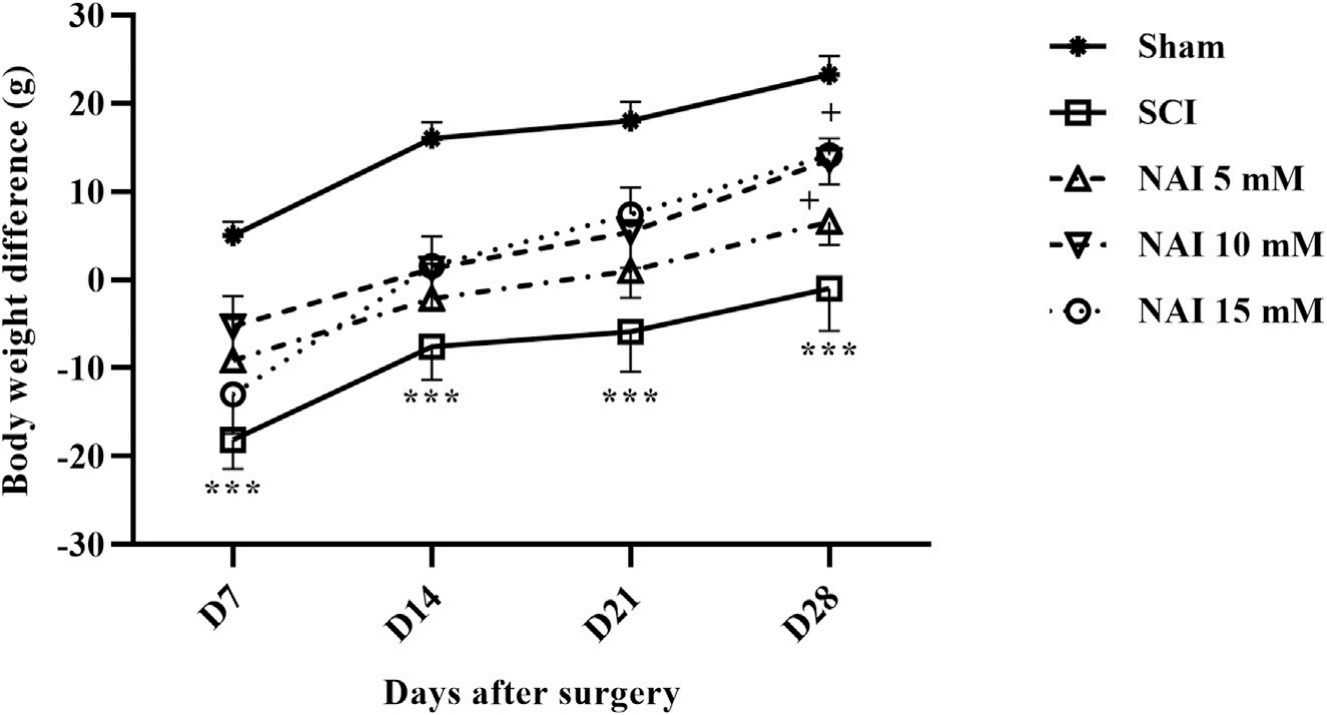
The impact of NAI on changes in body weight in rats undergoing compression SCI. The data presented are expressed as mean ± SEM (*n* = 7). ^***^ *p* < 0.001 vs. sham group and ^+^ *p* < 0.05 vs. SCI group. SCI: spinal cord injury; NAI: naringin (10 µL).

According to the findings, on the 7^th^ day after surgery, only the sham group showed weight gain, while the other groups experienced weight loss. The damaged group (SCI) had a more severe weight loss compared to others (*p* < 0.001. Figure 7). However, from the 7^th^ day to the 28^th^ day, there was an overall upward trend in weight changes. The group receiving NAI showed a steeper slope of weight gain compared to the SCI group, indicating that the animals in this group had a better weight gain process (*p* < 0.05).

## 4 Discussion

In the current study, we highlighted the effects of NAI on sensory-motor impairment following compression SCI in rats. The results indicated that NAI reduced mechanical pain, cold allodynia, and heat hyperalgesia, and additionally improved motor dysfunction following SCI. From the mechanistic point, NAI showed an increase in the activity of anti-inflammatory MMP-2, while decreasing inflammatory MMP-9. NAI also increased antioxidative glutathione and catalase and decreased serum nitrite levels. Of histopathological analysis, our results showed that administering NAI significantly decreased spinal tissue lesions, and increased neuronal survival in dorsal (sensory neurons) and ventral (motor neurons) horns of the spinal cord. As another result of our study, NAI attenuated weight changes in rats after SCI.

SCI is a complex condition that leads to various pathophysiological processes, including oxidative stress and inflammation. These processes contribute to secondary damages and worsen the outcomes of SCI. Oxidative stress occurs when there is a mismatch between the generation of ROS and the body’s capacity to detoxify those using antioxidants. After SCI, a boost in ROS production occurs, potentially causing cellular damage and triggering apoptosis. Glutathione and catalase are two important antioxidants involved in combating oxidative stress and are important antioxidants that help counteract ROS. Catalase plays a crucial role in safeguarding cells from oxidative damage by catalyzing the decomposition of hydrogen peroxide into water and oxygen. In SCI, the depletion of glutathione occurs because of heightened oxidative stress, which in turn intensifies the injury. Besides, catalase activity is often reduced after SCI, accumulating hydrogen peroxide and increasing oxidative stress (Jia et al., 2012; Bedreag et al., 2014). On the other hand, in pathological conditions like SCI, excessive nitric oxide production leads to nitrosative stress and tissue damage. This can result in the formation of peroxynitrite, a harmful molecule, through reactions with superoxide. The imbalance between ROS and antioxidant defenses causes oxidative stress and neuroinflammation, contributing to the progression of SCI (Conti et al., 2007). In the context of SCI, oxidative stress can cause damage to cellular components and exacerbate tissue injury. Therefore, maintaining adequate levels of glutathione, catalase, and nitric oxide is crucial to minimize oxidative damage and promote healing in the spinal cord (Espinosa-Diez et al., 2015; Zuo et al., 2023).

MMPs are enzymes that break down extracellular matrix proteins and are involved in inflammation. Excessive MMP activity can lead to tissue damage, disrupt the blood-spinal cord barrier, and increase oxidative stress by producing ROS. Some MMPs also degrade antioxidant enzymes like superoxide dismutase and catalase, impairing the body’s ability to counteract ROS. Targeting MMP-9 for inhibition has been proposed as a potential therapeutic strategy for SCI, showing promise in reducing tissue damage and enhancing functional recovery in animal models (Yu et al., 2008; Zhang et al., 2011). While MMP-9 is related to elevated inflammation and tissue damage, MMP-2 is linked to tissue repair and remodeling (Gravandi et al., 2024).

Indeed, treatment of SCI with currently approved drugs, such as methylprednisolone, has shown limited long-term efficacy and carries a high risk of complications (Sultan et al., 2020). Therefore, investigating and discovering the potential of new compounds that can bring more effectiveness with fewer side effects is highly needed. Meanwhile, herbal medicines have been used for centuries in traditional medicine systems and are known for their diverse chemical constituents and therapeutic properties (Firenzuoli and Gori, 2007). Moreover, herbal medicines are generally considered to have fewer side effects compared to synthetic drugs, as they are derived from natural sources. Herbal medicines often contain several active compounds that can affect several mechanisms simultaneously (Karimi et al., 2015). Since neurodegenerative diseases such as SCI are often complex and multi-mechanistic pathological processes (Anjum et al., 2020), it seems that these compounds can be a suitable therapeutic approach. NAI is one of the most important natural products, which has many therapeutic and medicinal effects, including antioxidant, anti-inflammatory, anti-angiogenic, anti-diabetic, anti-cancer, and antibacterial effects (Zaidun et al., 2018; Sharma et al., 2021; Motallebi et al., 2022).

Additionally, targeting oxidative stress pathways such as glutathione, and catalase may help mitigate the effects of MMP activity and reduce neurological disorders (Lee et al., 2011). In this regard, our results indicated that NAI treatment was effective at all three doses in lowering nitrite levels and enhancing glutathione activity after SCI. Notably, the 10 mM dose of NAI demonstrated greater efficacy in promoting catalase activity compared to the other doses which confirmed the hormesis theory based on reverse u-shaped dose-response effectiveness (Fakhri et al., 2022b). Our results also indicated that the administration of NAI had a direct impact on the modulation of MMP levels. In this instance, the effectiveness of the 5 and 15 mM doses was limited, whereas the 10 mM dose was associated with significant effects. Consequently, the control of inflammation and oxidative stress resulted in a notable enhancement of sensory-motor function. Previously, Lee and colleagues reported that NAI regulated MMP-9 expression (Lee et al., 2009). A study demonstrated that NAI showed positive effects on motor performance and neuroprotection in the context of vanadium-induced neurotoxicity. They mentioned that these effects were achieved by reducing oxidative stress, inflammation, and apoptosis (Adekeye et al., 2022). NAI has shown potentials in protecting dopaminergic neurons in Parkinson’s disease models by increasing neurotrophic factors such as glial cell line-derived neurotrophic factor (GDNF) and reducing inflammation. This neuroprotection could preserve motor function over time (Jung and Kim, 2014). Our tissue analysis demonstrated that NAI not only enhances sensory and motor function but also effectively preserves the integrity of spinal cord tissue and the neurons in both the dorsal and ventral horns. All three doses effectively reduced lesion volume from the first week. However, the impact of NAI on neuronal preservation became more pronounced starting from the third week. Although the 5 mM dose showed less efficacy, both the 10 and 15 mM doses exhibited substantial positive effects. Reports have also shown that NAI has a positive impact on neuron recovery following damage. In addition, it helps to preserve neurons and protects them from diseases of the central nervous system (Jung et al., 2014; Emran et al., 2022).

On the other hand, and in comparison to other models of SCI, compression SCI possesses minor limitations, similar to those in SCI patients and is more suitable for translational research (Cregg et al., 2014). Highlighting major limitations in pre-clinical SCI research protocols, SCI models usually employ young adult animals, while clinically engaging individuals with a wide age range (Stewart et al., 2022). Possible partial (not complete) damage of neural tracts and inability to directly apply long-distance axon regeneration are other limitations of pre-clinical studies. Besides, preclinical models of SCI are usually done during 4 weeks of follow-up with a limited number, which is not in accommodation with clinical cases with long-lasting complications. Larger volumes of human gray matter also need more time toward reinnervation. It slowdowns the spontaneous recovery procedure in humans after SCI (Bagheri Bavandpouri et al., 2024). Finally, the pharmacokinetic limitations in the use of phytochemicals urge the need for providing novel drug delivery systems with higher efficacy and lower side effects (Fakhri et al., 2022a).

## 5 Conclusion

NAI showed promising effects in reducing different types of pain, improving motor activity, and attenuating weight gain through anti-inflammatory, antioxidant, and neuroprotective roles. Future research should focus on long-lasting preclinical reports, employing different animal models and different routes of drug administration. Additionally, providing novel delivery systems and more pre-clinical reports are appreciated to find the most suitable formulation and evaluate detailed mechanistic pathways of NAI against SCI. Besides, well-controlled clinical trials on individuals receiving NAI are needed to confirm the pre-clinical studies.

## Data Availability

The raw data supporting the conclusions of this article will be made available by the authors, without undue reservation.
